# Diversity and function of maize pollen coat proteins: from biochemistry to proteomics

**DOI:** 10.3389/fpls.2015.00199

**Published:** 2015-03-30

**Authors:** Fangping Gong, Xiaolin Wu, Wei Wang

**Affiliations:** State Key Laboratory of Wheat and Maize Crop Science, Collaborative Innovation Center of Henan Grain Crops, College of Life Science, Henan Agricultural UniversityZhengzhou, China

**Keywords:** pollen coat proteins, maize, pollen–stigma interaction, pollen germination, proteomics

## Abstract

Maize (*Zea mays* L.) is globally cultivated as one of the most important grain crops. As a wind-pollinated species, maize produces a large quantity of pollen grains that heavier and larger compared to *Arabidopsis*. Maize is an important model plant in pollen biology of monocots. The pollen coat, the outermost layer of pollen, plays a vital role in pollen–stigma interactions and successful fertilization. Pollen coat proteins (PCPs), which confer species specificity, are required for pollen adhesion, recognition, hydration, and germination on the stigma. Thus, PCPs have attracted intensive research efforts in plant science for decades. However, only a few PCPs in maize have been characterized to date, whereas the functions of most maize PCPs remain unclear. In this review, we summarize the current knowledge of maize PCPs with regard to protein constituents, synthesis and transport, and functions by comparison with the model plant *Arabidopsis thaliana* and *Brassica* plants. An understanding of the comprehensive knowledge of maize PCPs will help to illuminate the mechanism by which PCPs are involved in pollen–stigma interactions in maize and other crop plants.

## Introduction

**FIGURE 1:**
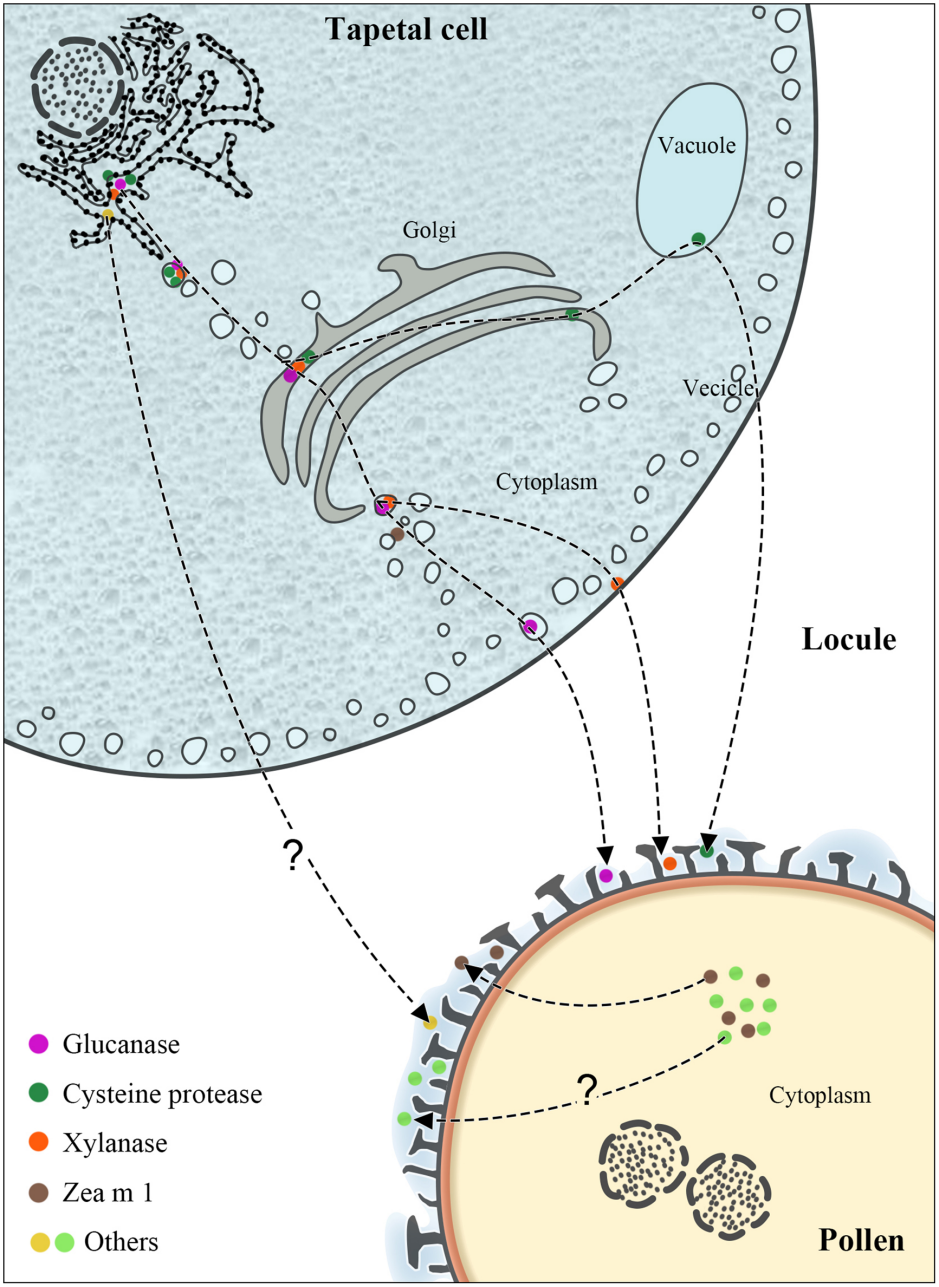
The synthesis and transport pathways of maize PCPs. The figure illustrates the characterized or possible transport pathways of maize PCPs from tapetum or pollen interior to the pollen coat.

The pollen coat, also called pollenkitt (Dobson, 1988) and tryphine (Murphy and Ross, 1998), is the outermost layer of pollen (**Figure 1**). Pollenkitt is most common in angiosperms, whereas tryphine refers to the pollen coat in insect-pollinated Brassicaceae plants (Pacini and Hesse, 2005). Despite the difference in the constituent and formation, both types of pollen coats originate from the anther tapetum and share some functions. The pollen coat, which confers species specificity, is composed of lipids, proteins, pigments, and aromatic compounds (Bih et al., 1999; Mayfield et al., 2001; Murphy, 2006), fills the sculptured cavities of the exine (Heslop-Harrison, 1968; Chay et al., 1992) and thus is highly heterogeneous and extremely hydrophobic. The study of pollen coat constituents dates back to the 1960s (Heslop-Harrison, 1968). The constituents of the pollen coat, especially pollen coat proteins (PCPs), are thought to play vital roles in aiding pollen–stigma recognition, adhesion, and hydration and pollen initial germination on the stigma (Doughty et al., 1993, 1998; Suen and Huang, 2007; Dresselhaus and Franklin-Tong, 2013). For example, SP11/SCR was found to determine pollen S-specificity in the self-incompatibility of *Brassica* species (Cabrillac et al., 2001; Shiba et al., 2001); in addition, xylanase facilitates maize pollen tube penetration into the silk via enzymatic xylan hydrolysis (Suen and Huang, 2007). Therefore, PCPs have attracted intensive research efforts in plant science for decades, especially in Brassicaceae plants (e.g., *Arabidopsis* and *Brassica napus*). However, only a few studies on PCPs in crop plants (e.g., maize and rice) are available to date.

Maize (*Zea mays*), belonging to the Poaceae family, is one of the most important cereal crops worldwide, and the completion of the maize B73 genome sequence has greatly promoted the progress of maize proteomics. Compared to *Arabidopsis*, maize pollen grains are heavier and larger (150–500 ng, 60–125 μm in diameter; Kalinowski et al., 2002), and adequate amounts of maize pollen grains can easily be obtained in high purity for biological studies. Therefore, maize serves as an important model for exploring the mechanisms of pollen germination, pollen–sigma interaction and pollen tube growth in monocots.

To our knowledge, only a few major PCP constituents in maize, e.g., cysteine protease, β-expansin (Zea m 1), xylanase and β-glucanase (Bih et al., 1999; Suen et al., 2003; Wang et al., 2006; Li et al., 2012), have been identified and characterized using biochemical and molecular techniques, whereas most PCPs in maize remain to be characterized. Proteomics is a powerful tool for analyzing complex mixtures of proteins and identifying biomarkers. For the first time, we have recently identified the overwhelming majority of the maize PCPs via a gel-based proteomic technique (Wu et al., 2015). Based on informatics analyses, many maize PCPs have been proposed to interact with stigma surface constituents.

In this review, the current knowledge of maize PCPs, including the protein constituents, synthesis and transport, and functions, are summarized. By comparison with the model plant *Ar. thaliana* and *Brassica* plants, we highlight the specificity of maize PCPs and the potential roles of specific PCPs in pollen–stigma interactions during early pollen germination.

## The Isolation of Maize PCPs

The lipidic pollen coat can be readily removed by treatment with organic solvents such as cyclohexane, diethyl ether, and chloroform (Doughty et al., 1993; Murphy and Ross, 1998). PCPs in such a pollen coat preparation are then extracted and purified using detergent-containing buffers. The extraction effects of different organic solvents on PCPs have been evaluated. Carbon tetrachloride and chloroform were reported to extract relatively more species of maize PCPs compared to six other organic solvents (hexane, heptane, cyclohexane, benzene, diethyl ether, and methanol; Bih et al., 1999). However, due to its high protein extractability and low pollen hydration rates, cyclohexane was found to be the best solvent (out of 24 organic solvents, ranging from non-polar to polar) for Bermuda grass (*Cynodon dactylon*) pollen coat extraction (Bashir et al., 2013).

Previously, three major maize PCPs (endoxylanase, β-glucanase, and cysteine protease; Bih et al., 1999; Suen et al., 2003) and a small amount of Zea m 1 (Wang et al., 2006) were extracted from a pollen coat preparation using diethyl ether, and we recently used chloroform to extract maize PCPs for a proteome analysis (Wu et al., 2015).

## The Constituents of Maize PCPs

At present, the known PCPs in maize include a total of 14 protein species, including allergens, hydrolases, and other proteins (**Table 1**). With the exception of the previously characterized endoxylanase, β-glucanase, cysteine protease and β-expansin 1 (Zea m 1), most PCPs have been identified via gel-based proteomics (Wu et al., 2015). Endoxylanase, β-glucanase, Zea m 1, and cysteine protease are present in high abundance in the maize pollen coat. In addition, many maize PCPs (e.g., profilin, exopolygalacturonase, and ABA-induced caleosin) exist in several isoforms.

**Table 1:** The summary of identified PCPs and their functions in maize.

Description	Protein ID^a^	Extraction solvents	Identification method	Relative level	Molecular function^b^	Biological process	Reference
**Hydrolase**
Cysteine protease	B5KVP9	Diethyl ether; chloroform	SDS-PAGE, immunoblotting; 2-DE, MS/MS	+++	Cysteine-type peptidase activity	Involvement in tapetal programmed cell death; possibly involvement in protein hydrolysis and/or interactions on stigma surface during pollen germination	Suen et al. (2003), Li et al. (2012), Zhang et al. (2014), Wu et al. (2015)
Endoxylanase	Q9ZTB8	Diethyl ether; chloroform	SDS-PAGE, immunoblotting; 2-DE, MS/MS	+++	Hydrolase activity, hydrolyzing *O*-glycosyl compounds	Facilitating pollen tube penetration into silk via enzymatic xylan hydrolysis	Bih et al. (1999), Suen and Huang (2007), Wu et al. (2015)
β-Glucanase	Q6VB92	Diethyl ether	SDS-PAGE, immunoblotting; 2-DE, MS/MS	+++	Hydrolase activity, hydrolyzing *O*-glycosyl compounds	Facilitating pollen tube penetration into silk via enzymatic glucan hydrolysis	Suen et al. (2003)
Putative subtilase	C5XXJ2	Chloroform	2-DE, MS/MS	+	Serine-type endopeptidase activity	Not assigned	Wu et al. (2015)
**Allergen**
β-Expansin-10 (Zea m 1)	Q1ZYQ8	Chloroform	2-DE, MS/MS	+++	Loosening cell wall	Facilitating pollen separation, penetration into stigma and pollen tube growth	Valdivia et al. (2009), Wu et al. (2015)
β-Expansin 1 (Zea m 1)	P58738	Diethyl ether	SDS-PAGE, immunoblotting; 2-DE, MS/MS	+++	Loosening cell wall	Facilitating pollen separation, penetration into stigma and pollen tube growth	Wang et al. (2006), Valdivia et al. (2009)
Phl p 2 precursor homolog (Zea m 2)	B6TDY2	Chloroform	2-DE, MS/MS	+++	Not assigned	Not assigned	Wu et al. (2015)
Profilin-1 (Zea m 12)	P35081	Diethyl ether; chloroform	SDS-PAGE, immunoblotting; 2-DE, MS/MS	++	Binding to actin and affecting the structure of the cytoskeleton	Involvement in cytoskeleton organization inside the pollen tube; possibly playing a protective role in pollen coat	Suen et al. (2003), Wu et al. (2015)
Profilin-2 (Zea m 12)	P35082	Diethyl ether; chloroform	SDS-PAGE, immunoblotting; 2-DE, MS/MS	++	Binding to actin and affecting the structure of the cytoskeleton	Involvement in cytoskeleton organization inside the pollen tube; possibly playing a protective role in pollen coat	Suen et al. (2003), Wu et al. (2015)
Profilin-3 (Zea m 12)	P35083	Diethyl ether; chloroform	SDS-PAGE, immunoblotting; 2-DE, MS/MS	++	Binding to actin and affecting the structure of the cytoskeleton	Involvement in cytoskeleton organization inside the pollen tube; possibly playing a protective role in pollen coat	Suen et al. (2003), Wu et al. (2015)
Exopolygalacturonase (Zea m 13)	P26216	Diethyl ether; chloroform	SDS-PAGE, immunoblotting; 2-DE, MS/MS	+++	Galacturan 1,4-α-galacturonidase activity	Facilitating tube advance by hydrolyzing the pectin between adjacent cells in the transmitting track	Suen et al. (2003), Wu et al. (2015)
**Other PCPs**
ABA-induced caleosin	B6TKU1	Chloroform	2-DE, MS/MS	+++	Calcium ion binding	Possibly involvement in pollen–stigma communication	Wu et al. (2015)
Rho GDP-dissociation inhibitor 1 (RhoGDI 1)	B6TH58	Chloroform	2-DE, MS/MS	++	Rho GDP-dissociation inhibitor activity	Involvement in pollen tube tip growth	Fu et al. (2001), Chen et al. (2003), Wu et al. (2015)
Ras-related protein Rab-2-A (RAB2A)	P49103	Chloroform	2-DE, MS/MS	+	Protein transport	Involvement in pollen tube tip growth	de Graaf et al. (2005), Szumlanski and Nielsen (2009), Wu et al. (2015)

*^a^Protein ID in UniProtKB (http://www.uniprot.org/)*

*^b^Molecular function annotated in UniProtKB (http://www.uniprot.org/)*.

The proteins identified in maize pollen coat are greatly different from those in Brassicaceae plants. In *A. thaliana*, 10 PCPs (>10 kDa) have been identified, including two kinases, one caleosin-like proteins, two lipase proteins, and five oleosins (Mayfield et al., 2001). In *B. napus*, 12 PCPs have been identified, most of which are oleosin isoforms (Murphy, 2006). In particular, the amphipathic oleosin, originating from storage tapetosomes in tapetum cells (Wu et al., 1997; Ting et al., 1998), has been demonstrated to be absent from the maize pollen coat (Li et al., 2012).

This largely difference in PCPs between maize and Brassicaceae plants is largely related to the physiological property and formation, and fundamentally the species specificity. Indeed, the pollen coat constituents in wind-pollinated species such as maize are quite different from that in insect- or self-pollinated species. *Brassica* and *Arabidopsis* are insect- or self-pollinated species, and their pollen has a thick coat, which is sticky and contains abundant lipids. In contrast, maize pollen has a thinner pollen coat that is non-sticky and contains a reduced amount of lipids (Bih et al., 1999). In addition, maize PCPs can originate from the tapetum or the pollen interior.

## Synthesis and Transport of Maize PCPs

The tapetum, forming the innermost sporophytic cell layer of the anther and enveloping the developing pollen (**Figure 1**), plays a central role in pollen coat and exine formation (Ariizumi and Toriyama, 2011; Liu and Fan, 2013). In *Arabidopsis* and *Brassica* species, the tapetum is packed with two predominant storage organelles: elaioplasts and tapetosomes (Hsieh and Huang, 2007; Li et al., 2012; Quilichini et al., 2014a,b). The constituents of the pollen coat, such as steryl esters, lipids, alkanes, lipid-associated proteins, oleosin proteins, and flavonoids, are mainly derived from the elaioplasts and tapetosomes. However, electron microscopy studies have shown that at a late developmental stage, maize tapetum cells do not possess elaioplasts and tapetosomes (Li et al., 2012; Liu and Fan, 2013), indicating that maize PCPs synthesized in the tapetum may be delivered to the pollen coat via other mechanisms.

Although relatively more maize PCPs have been reported of late, our knowledge about their exact origin and transport routes is limited. Glucanase, xylanase, and cysteine protease have been demonstrated to be synthesized in the adjacent tapetum and transported via the endoplasmic reticulum (ER), Golgi, vacuoles, and vesicles (**Figure 1**); some remain in the tapetum, eventually depositing in the sculptured cavities of the pollen exine upon programmed cell death of the tapetum (Li et al., 2012). The synthesis of glucanase and xylanase begins at the middle stage of anther development, and these enzymes are then stored in vesicles and the cytosol, respectively; in contrast, cysteine protease first emerges at the late stage and is stored in vacuoles. Both glucanase and cysteine protease contain an ER-targeting signal peptide. Xylanase is initially synthesized via a tapetum mRNA with a long 5′ leader (Bih et al., 1999) as a 60-kDa precursor, which is then converted to the active 35-kD xylanase (Wu et al., 2002).

Zea m 1, together with Cyn d 1, Sor h 1, Lol p 1, and Phl p 1, are pollen-specific group-1 allergens (Valdivia et al., 2007; Bashir et al., 2013). Phl p 1 is mainly present in the pollen intine (Grote et al., 1994; Behrendt et al., 1999) and also in the pollen coat and cytosol of pollen vegetative cells (Staff et al., 1990). By immunoelectron microscopy, Wang et al. (2006) found Zea m 1 in the pollen coat fraction, in the tectum and the foot layer of the exine. Besides, a substantial amount of β-expansin (Zea m 1) was found localized in pollen interior (Suen et al., 2003; Wang et al., 2006). Moreover, two allergens profilin (Zea m 12) and exopolygalacturonase (Zea m 13) were found to be easily released from pollen into aqueous solution (Suen et al., 2003), implying a pollen surface localization. Our recent work showed that profilin and exopolygalacturonase exist in the coat of maize pollen (Wu et al., 2015). Thus, it is possible that these allergens in the maize pollen coat may be synthesized within the pollen interior (**Figure 1**).

Sporopollenin is the main component of the exine and is exported from intact tapeta during the tetrad stage to the early bicellular pollen stage (Quilichini et al., 2014a,b). Within this time frame, lipid transfer proteins are abundant in the locule fluid (Huang et al., 2013), which is in direct contact with both the tapetal cells and developing pollen grains. Interestingly, lipid transfer proteins are also found in the pollen coat after tapetum programmed cell death (Huang et al., 2013; Quilichini et al., 2014a). Therefore, it is speculated that some PCP constituents appear in the exine and pollen coat via sporopollenin traffic from the tapeta to the developing pollens in flowering species with secretory tapeta (Quilichini et al., 2014a). However, the exact transport pathway of many PCPs in maize still needs to be verified.

## The Functions of Maize PCPs

As maize pollen grains usually germinate within 5 min when landing on the stigma (Heslop-Harrison, 1979), it is difficult to systematically study the functions of maize PCPs in such a short time window. To elucidate the functions of maize PCPs during pollen–stigma interaction, pollen germination and tube growth, mutant pollen grains were generated using antisense or RNAi techniques to measure the resulting phenotype of pollen grains *in vitro* and *in vivo*. Among the identified maize PCPs, only three (xylanase, β-glucanase, and β-expansin 1) have been functionally characterized in maize, though the homologs of other PCPs have been studied in other tissues or species (Bashir et al., 2013; Zhang et al., 2014).

According to their synthesis pathways, the maize PCPs discussed herein can be divided into three groups. Group 1 includes tapetum-synthesized hydrolases, e.g., xylanase, β-glucanase, cysteine protease, and subtilase. For successful germination, pollen gains need to overcome the mechanical resistance from a thin protein layer and the polysaccharide-rich wall in the style (stigma). It may be the result of evolution that these enzymes become part of pollen coats to facilitate the penetration of pollen tube into the style tissues during sexual reproduction.

After tapetum cells rupture, their contents are scattered and enveloped by the pollen. Xylanase on the pollen coat, together with other hydrophilic components, initially helps to provide sufficient water along the pollen surface to the aperture for pollen germination. At the same time, xylanase begins the hydrolysis of carpel wall xylan to create an opening for pollen tube entry (Suen and Huang, 2007). Along with coat xylanase, coat β-glucanase also hydrolyzes the stigma wall during pollen germination (Suen et al., 2003). Pollen coat β-glucanase is notably different from the enzyme that hydrolyzes the callose wall of the microspore tetrad (Suen et al., 2003; Takeda et al., 2004). After the pollen tube penetrates into the stigma, β-glucanases appear to hydrolyze these glucans, playing an important role in the regulation of pollen tube elongation (Takeda et al., 2004). In addition, xylanase and cysteine protease on the pollen coat of Bermuda grass have dual functions: IgE-binding capacity and proteolytic activity, which disrupts the integrity of the human airway epithelial cell barrier (Bashir et al., 2013). It would be interesting to examine the potential proteolytic activity of both PCPs on the surface of maize stigma cells.

Cysteine protease exists widely in animals, plants and parasites. In *Arabidopsis*, cysteine protease is a key executor involved in tapetal programmed cell death and thus regulates pollen development (Zhang et al., 2014). In maize, cysteine protease is the only known tapetum protease that appears at a very late stage of anther development (Li et al., 2012). Based on its substantial abundance in maize pollen coat (Wu et al., 2015), cysteine protease may function by interacting with (or hydrolyzing) a thin layer of proteins on the stigma surface during pollen germination.

Group 2 comprises pollen-synthesized proteins, including various allergens, such as Zea m 1 (β-expansins 1, 10), Zea m 12 (profilin), and Zea m 13 (exopolygalacturonase). Profilin is the main monomer actin-binding protein involved in cytoskeleton organization and is essential for tip growth of plant cells (Vidali et al., 2007). In addition to the existence in cytosol, profilin exists in plasma membrane (Marmagne et al., 2007) and cell wall (Van Damme et al., 2004). In maize pollen coat, profilin may strengthen the protective role of the coat via binding phospholipids.

After pollen landing on the stigma, these pollen-synthesized proteins may be released to assist in pollen tube penetration into the stigma and in the subsequent hydrolysis and modulation of the carpel interior wall. For example, polygalacturonase (Zea m 13) hydrolyzes the pectin between adjacent cells in the transmitting track and facilitates tube advance (Suen et al., 2003). Zea m 1 (β-expansin) can loosen stigmatic cell walls and aid pollen tube penetration of the stigma (Cosgrove et al., 1997). A reduction in Zea m 1 level in maize pollen by insertional mutation was found to greatly affect pollen tube growth rates and thus pollen competition *in vivo* (Valdivia et al., 2007). Moreover, the pollen deficient in Zea m 1 gene expression tended to form large aggregates, leading to poor pollen dispersal upon anther dehiscence, and the emerging pollen tubes had difficulty entering the style (Valdivia et al., 2009). So, Zea m 1 is required for pollen separation and stigma penetration and plays an important role in determining the outcome of pollen competition *in vivo* for access to ovules.

Group 3 includes various proteins, such as ABA-induced caleosin, rho GDP-dissociation inhibitor 1 (RhoGDI 1) and ras-related protein Rab-2-A (RAB2A). Caleosin has been detected in the pollen coats of *A. thaliana* (Mayfield et al., 2001), *B. napus* (Murphy, 2006), and maize (Wu et al., 2015). Structurally, caleosin contains an N-terminal region with a single Ca^2+^-binding EF-hand domain, a central hydrophobic region, and a C-terminal region with several putative protein kinase phosphorylation sites. These signaling-related motifs suggested that caleosin may play a role in pollen–stigma communication. RhoGDI is involved in Rac/Rop GTPase-regulated pollen tube growth through prevents the formation of transversal actin bands (Fu et al., 2001; Chen et al., 2003). Similarly, RAB2A belongs to the rab GTPase family that is important for pollen tube tip growth (de Graaf et al., 2005; Szumlanski and Nielsen, 2009). Thus, the biological functions of RhoGDI 1 and RAB2A in maize pollen coat are worth to be studied.

## Conclusion and Perspectives

Despite the diversity of known maize PCPs, only a few PCPs have been functionally characterized. Indeed, the functions of other PCPs, especially those in large abundance in the maize pollen coat, remain unclear, though some functional clues are available through informatics analyses. The precise biological role of uncharacterized maize PCPs can be established in the future through the analysis of transgenic mutant pollen by gain-of-function or loss-of function approaches. An understanding of the comprehensive knowledge of maize PCPs will help to illuminate the mechanism by which PCPs are involved in pollen–stigma interactions in maize and other crop plants.
